# The Stroke Oxygen Pilot Study: A Randomized Controlled Trial of the Effects of Routine Oxygen Supplementation Early after Acute Stroke—Effect on Key Outcomes at Six Months

**DOI:** 10.1371/journal.pone.0059274

**Published:** 2013-06-03

**Authors:** Khalid Ali, Anushka Warusevitane, Frank Lally, Julius Sim, Sheila Sills, Sarah Pountain, Tracy Nevatte, Martin Allen, Christine Roffe

**Affiliations:** 1 Academic Department of Geriatrics, Brighton and Sussex Medical School, Brighton, United Kingdom; 2 Stoke Stroke Research Group, North Staffordshire Combined Healthcare Trust, Stoke-on-Trent, United Kingdom; 3 Institute for Science and Technology in Medicine, Keele University, Staffordshire, United Kingdom; 4 Health Services Research Unit, Keele University, Staffordshire, United Kingdom; 5 Department of Respiratory Medicine, University Hospital of North Staffordshire, Stoke-on-Trent, United Kingdom; Julius-Maximilians-Universität Würzburg, Germany

## Abstract

**Introduction:**

Post-stroke hypoxia is common, and may adversely affect outcome. We have recently shown that oxygen supplementation may improve early neurological recovery. Here, we report the six-month outcomes of this pilot study.

**Methods:**

Patients with a clinical diagnosis of acute stroke were randomized within 24 h of admission to oxygen supplementation at 2 or 3 L/min for 72 h or to control treatment (room air). Outcomes (see below) were assessed by postal questionnaire at 6 months. Analysis was by intention-to-treat, and statistical significance was set at *p*≤0.05.

**Results:**

Out of 301 patients randomized two refused/withdrew consent and 289 (148 in the oxygen and 141 in the control group) were included in the analysis: males 44%, 51%; mean (SD) age 73 (12), 71 (12); median (IQR) National Institutes of Health Stroke Scale score 6 (3, 10), 5 (3, 10) for the two groups respectively. At six months 22 (15%) patients in the oxygen group and 20 (14%) in the control group had died; mean survival in both groups was 162 days (*p* = 0.99). Median (IQR) scores for the primary outcome, the modified Rankin Scale, were 3 (1, 5) and 3 (1, 4) for the oxygen and control groups respectively. The covariate-adjusted odds ratio was 1.04 (95% CI 0.67, 1.60), indicating that the odds of a lower (i.e. better) score were non-significantly higher in the oxygen group (*p* = 0.86). The mean differences in the ability to perform basic (Barthel Index) and extended activities of daily living (NEADL), and quality of life (EuroQol) were also non-significant.

**Conclusions:**

None of the key outcomes differed at 6 months between the groups. Although not statistically significant and generally of small magnitude, the effects were predominantly in favour of the oxygen group; a larger trial, powered to show differences in longer-term functional outcomes, is now on-going.

**Trial Registration:**

Controlled-Trials.com ISRCTN12362720; Eudract.ema.europa.eu 2004-001866-41

## Introduction

Hypoxia is common after acute stroke and may have significant adverse effects on the ischaemic brain [1]–[3]. Hypoxia is particularly likely to occur at times when the patient tends not to be observed so closely, e.g. during the head scan, during transfer from the emergency department to the ward, and at night [3]. In an acute stroke unit, where oxygen saturation was assessed every six hours, 52% of stroke patients with normal oxygen saturation in the day had five minutes or more of hypoxia (oxygen saturation lower than 90%) at night on analysis of continuous pulse oximetry; 23% were hypoxic for more than 30 min, and 15% for more than 1 h [4]. While continuous pulse oximetry is available in most, if not all, UK stroke units, it is marred by frequent false alarms due to displacement of the finger probe. Reliable detection of hypoxia by this means requires a quasi-intensive care environment with a nurse free to check every desaturation alarm immediately. Prompt and effective treatment of hypoxia may be one of the reasons why patients nursed on a stroke unit have better outcomes. Such patients are more likely to receive oxygen than on a non-specialized general ward [5]. Treating all episodes of hypoxia with supplemental oxygen has been identified as one of three key processes associated with better outcome in acute stroke care [6]. Routine oxygen supplementation during the first few days after the stroke, when the ischaemic brain is most vulnerable, could be an effective method of reducing the hypoxic burden and improving outcome. The aim of the Stroke Oxygen Pilot Study is to determine whether low-flow oxygen at a rate of 2 or 3 L/min, dependent on baseline oxygen saturation, with the intention to keep oxygen saturation within the normal range over a period of 72 hours, improves outcome after acute stroke. Week one results of the study suggest that early neurological recovery may be improved by oxygen supplementation [7]. In this paper we present functional and quality of life outcomes of this study at six months; the study is reported in line with the CONSORT statement [8].

## Methods

### Design, recruitment, intervention, and baseline assessments

The protocol for this trial and supporting CONSORT checklist are available as supporting information; see Checklist S1 and Protocol S1.

This is a randomized controlled single-blind pilot study of routine oxygen supplementation after acute stroke. Detailed methodology and neurological outcome at one week have previously been reported [7]. In this paper we present the long-term (six-month) outcomes. In short, adult patients with a clinical diagnosis of acute stroke as defined by the World Health Organization [9] were eligible for inclusion if they were admitted to the University Hospital of North Staffordshire within the preceding 24 hours, were able to give informed consent, or a relative was contactable and willing to give assent, and if there was no clear indication for or against oxygen treatment. Recognized indications for oxygen treatment were: oxygen saturation on air <90%, acute left ventricular failure, severe pneumonia, pulmonary embolus, and chronic respiratory failure treated with long-term oxygen at home. We excluded patients with contraindications to fixed-dose oxygen treatment at a rate of 2 or 3 L/min (e.g. type II respiratory failure), patients where stroke was not the primary clinical problem, and patients with other serious life-threatening illnesses likely to lead to death within a few months. As this was a pilot study, the sample size was not determined through a formal power calculation, but what was achievable within available resources.

Participants were randomized to two treatment arms: (1) Oxygen via nasal cannulae at a flow rate of 2 L/min if baseline oxygen saturation (SpO_2_) was greater than 93% or 3 L/min if baseline SpO_2_ was 93% or less for a period of 72 hours. (2) Control (oxygen only when clinically indicated). Randomization was by telephone or web portal access to a remote centre, and employed a computerized randomization algorithm. Heart rate, blood pressure, and SpO_2_ were assessed regularly (at least three times a day) as part of routine clinical care. Those participants who developed indications for oxygen, or needed a higher concentration of oxygen than the protocol prescribed, were given the appropriate concentration of oxygen by the treating clinician, irrespective of the treatment group. Baseline demographic and clinical details were established by interview and consultation of the medical notes and included the Oxfordshire Community Project Stoke Classification [10], oxygen saturation on room air, the National Institutes of Health Stroke Scale (NIHSS) [11] and the result of the computed tomography (CT) head scan. Using the ‘six simple variables’ (SSVs) in the SCOPE predictive model [13], [14], we also calculated two prognostic indices: (i) the probability of being free of dependency at six months (defined as a modified Rankin Scale (mRS) [12] less than 3) and (ii) the probability of survival at 30 days. The indices for this predictive model are derived from the patient's age and five dichotomous variables: living alone before stroke; independent before stroke; normal verbal response on the Glasgow Coma Scale [15]; ability to lift the arm against gravity; and ability to walk unaided.

### Six-month outcomes

Data were obtained using a postal questionnaire 6 months post stroke. Participants were invited to complete the questionnaire personally or with help from a family member or carer; this was recorded on the questionnaire. The primary outcome measure for this study was the mRS at 6 months [12]. This ordinal scale measures the degree of disability and dependence post stroke.

Other measures included the abbreviated 3-item Barthel Index [16], the EuroQol standardized quality of life scale [17], [18], and the Nottingham Extended Activities of Daily Living Scale (NEADL) [19]. The 3-item Barthel Index covers continence, bed to chair transfers, and mobility, with scores ranging from 0 (unable to do these activities) to 8 (able to do all independently). We used the formula ([total 3-item score]×2.39)+0.14 [16] to convert this to the more commonly used 10-item (20-point) Barthel Index [20]. The EuroQol describes quality of life in five dimensions (EQ-5D): mobility, self-care, usual activities, pain/discomfort and anxiety/depression. Each is scored as 1 (no problem), 2 (some problems), or 3 (not able/extreme). For analysis, scores from the five dimensions were combined into a single utility score. This score can be adjusted according to different population based weightings; here we used the weighting scheme derived for the UK [17], giving a possible range of scores from −0.59 to 1.00. The EQ-5D is accompanied by a visual analogue scale (EQ-VAS) on which participants self-rate their heath from 0 (worst imaginable health state) to 100 (best imaginable health state). The NEADL assesses the ability of participants to perform 20 extended activities of daily living, covering mobility, kitchen, domestic, and leisure activities. Each activity allows four responses: able to perform this alone easily (1), alone with difficulty (2), with help (3), and not all (4), giving a summated range of scores from 20 (able to do all activities easily) to 80 (unable to do any of the activities).

### Statistical analysis

Data were analysed on an intention-to-treat basis, and for each outcome the covariate-adjusted analysis was designated as the primary analysis; covariates were selected a priori on the basis of their clinical and prognostic importance. Missing values on the outcome measures were imputed through multiple imputation, under a missing-at-random assumption, using six imputed datasets. A sensitivity analysis was also performed using only cases with complete follow-up data [21].

Between-group differences on the primary outcome, the mRS, and the Barthel Index were analysed by proportional odds ordinal logistic regression (with the oxygen group as reference category), adjusting for the following covariates: age, sex, probability of being free from dependency at 6 months, oxygen saturation at randomization, type of stroke, and baseline values of the NIHSS. The Barthel Index and the NEADL were analysed through analysis of covariance, adjusting for the same covariates. Baseline data were not available for any of these outcome measures and no adjustment was therefore possible.

For 6-month survival, a log-rank test was performed for an unadjusted analysis. For an adjusted analysis, a Cox proportional-hazards regression model was used, with these covariates: age, sex, probability of 30-day survival, oxygen saturation at randomization, type of stroke, and baseline values of the NIHSS. The proportional hazards assumption was tested via Schoenfeld residuals and found to be tenable (*p* = 0.72).

Data were analysed in SPSS v20 (IBM, Armonk NY, USA) and imputations were performed in SOLAS 2 (Statistical Solutions, Cork, Ireland). Statistical significance was set at *p*≤.05 (two-tailed) and 95% confidence intervals (CIs) were calculated for all estimates.

### Ethical approval, trial registration, and consent

The protocol was approved by the North Staffordshire Research Ethics Committee on 06.10.2004 (ref: 04/Q2604/73). It is registered in the International Standard Randomized Controlled Trial Number Register (ISRCTN12362720) and the European Clinical Trials Database (EudraCT number 2004-001866-41). Written informed consent was sought from all study participants. Assent from the next of kin was accepted if the patient agreed to take part but was unable to give fully informed consent. Participants who were incompetent at the time of recruitment, but competent when followed-up at 1 week were asked to confirm consent. Patients who were recruited to the study by assent and refused consent at 1 week were withdrawn from the study. Requests for the trial data should be addressed to the corresponding author.

## Results

### Recruitment

**Figure 1:**
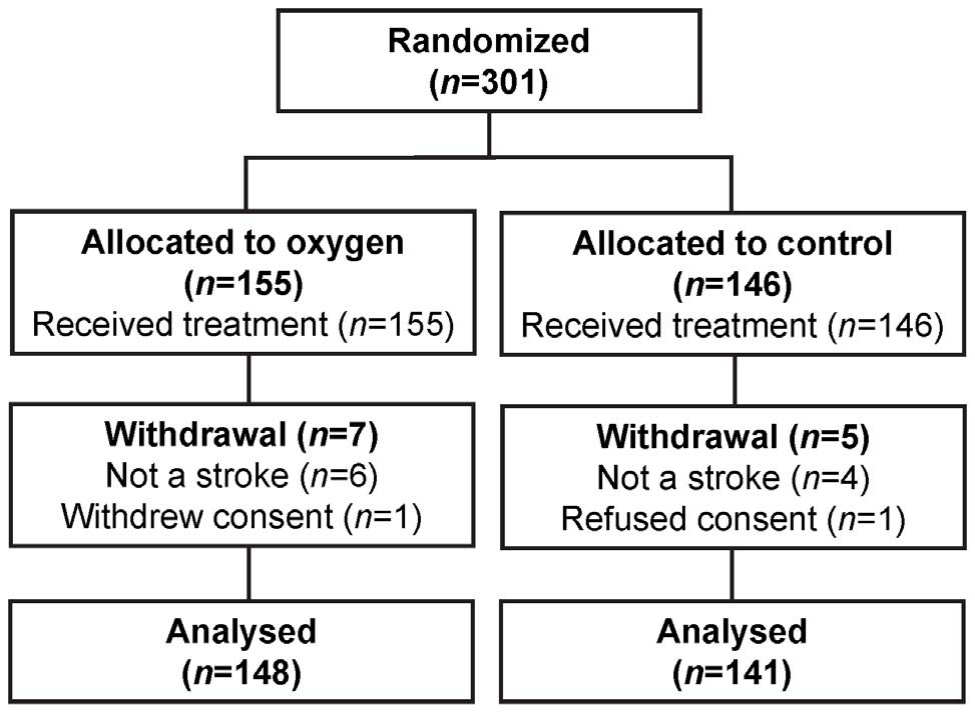
Flow of patients through the study.

The flow of patients through each stage of the study has previously been published for the week one results [7] and is the same for the main follow-up at 6 months (Figure 1). Three hundred and one patients were recruited to the study from October 2004 to April 2008. A total of 155 patients were allocated to oxygen and 146 to control. All randomized participants were subsequently given the allocated treatment. In 6 participants in the oxygen group and 4 in the control group, the initial clinical diagnosis of stroke was shown to be incorrect after further investigation (brain tumour *n* = 7, motor neurone disease *n* = 1, possible multiple sclerosis *n* = 1, no final diagnosis *n* = 1). One patient in each treatment group was excluded due to withdrawal of consent, leaving 148 and 141 subjects in the oxygen and control group to be considered in the final analysis.

### Baseline characteristics

Baseline clinical and demographic characteristics of the groups are shown in Table 1. Most variables were well balanced across groups, except for age, gender, probability of being free from dependency at 6 months, and baseline NIHSS, with the oxygen group having a higher mean age, more females, a lower probability of being free from dependency at 6 months, and a higher baseline median NIHSS score.

**Table 1:** Baseline characteristics.

	Oxygen (*n* = 148)	Control (*n* = 141)
**Demographic characteristics**		
Age in years; mean (SD)	73 (12)	71 (12)
Male gender; *n* (%)	65 (44)	72 (51)
**Prognostic factors**		
Living alone; *n* (%)	61 (41)	52 (37)
Independent in basic activities of daily living; *n* (%)	122 (82)	121 (86)
Normal verbal response; *n* (%)	102 (69)	92 (65)
Able to lift affected arm; *n* (%)	92 (62)	92 (65)
Able to walk; *n* (%)	20 (14)	21 (15)
Probability: non-dependent at 6 months; median (IQR)	0.19 (0.02, 0.55)	0.27 (0.03, 0.66)
Probability: 30-day survival; median (IQR)	0.23 (0.15, 0.40)	0.23 (0.13, 0.37)
**Concomitant medical problems**		
Ischaemic heart disease; *n* (%)	34 (23)	37 (26)
Left ventricular failure; *n* (%)	16 (11)	18 (13)
Atrial fibrillation; *n* (%)	34 (23)	19 (14)
Chronic obstructive pulmonary disease/asthma; *n* (%)	14 (10)	12 (9)
**Details of the stroke**		
Time (hh:mm) since stroke; mean (SD)	17:48 (8:46)	16:31 (8:08)
Hemiparesis; *n* (%)		
Right	59 (40)	61 (43)
Left	76 (51)	66 (47)
None	11 (7)	14 (10)
Aetiology; *n* (%)^a^		
Ischaemic stroke	136 (91)	121 (86
Haemorrhagic stroke	9 (6)	15 (11)
Not established	3 (2)	5 (4)
Total anterior circulation syndrome; *n* (%)	71 (51)	71 (51)
Glasgow Coma Scale score (3–15); (median, IQR)	15 (15, 15)	15 (15, 15)
NIHSS score (0–42); median (IQR)	6 (3, 10)	5 (3, 10)
Oxygen saturation at randomization; % mean (SD)	96.1 (1.9)	96.1 (2.0)

a Patients presenting with symptoms of stroke were defined as infarcts when the computed tomogram (CT) of the head showed no evidence of an alternative diagnosis.

A diagnosis of haemorrhage included intracerebral, subdural and subarachnoid haemorrhages. Aetiology could not be established in cases where a CT was not performed. SD = standard deviation; IQR = interquartile range; NIHSS = National Institutes for Health Stroke Scale.

### Who completed the 6 month questionnaire?

The questionnaire was completed in 112 and 111 participants (89% and 92% of survivors) in the oxygen and control groups respectively. Only just over a third completed the questionnaires unaided (Table 2). There were missing values on the modified Rankin scale (33 values), the EQ-5D (67 values) and EQ-VAS (112 values), the NEADL (96 values), and the Barthel Index (62 values).

**Table 2:** Completion of the six-month questionnaire.

	Oxygen	Control
	*n* = 126	*n* = 121
Participant completed it on his/her own	45 (35)	42 (35)
Completed with help from a relative/friend	18 (14)	21 (17)
Completed by a relative/friend	25 (20)	37 (31)
Completed by the researcher via phone, notes, clinic, ward	24 (20)	11 (9)
Question not answered	14 (11)	10 (8)

Data are number (%). Only survivors are included.

### The Modified Rankin Scale

**Figure 2:**
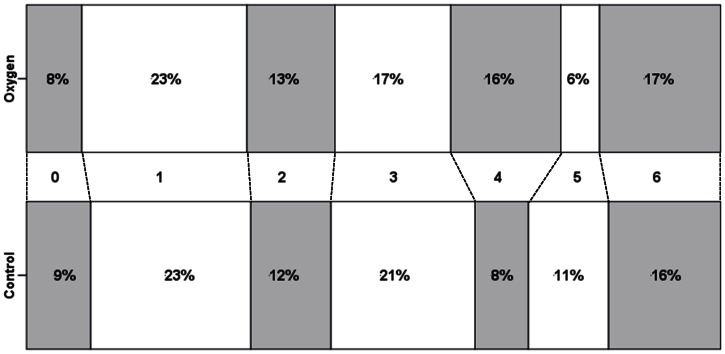
Distribution of modified Rankin Scale scores in each treatment group. Key. 0: No symptoms at all, 1: No significant disability despite symptoms, able to carry out all usual duties and activities, 2: Slight disability; unable to carry out all previous activities, but able to look after own affairs without assistance, 3: Moderate disability; requiring some help, but able to walk without assistance, 4: Moderately severe disability; unable to walk without assistance and unable to attend to own bodily needs without assistance, 5: Severe disability; bedridden, incontinent and requiring constant nursing care and attention, 6: Dead.

This scale was completed for 126 (85%) and 130 (92%) participants in the oxygen and control groups respectively. The number of participants independent at six months (mRS<3) was 56 (44%) and 58 (45%) in the oxygen and control groups respectively. The median (IQR) values on the mRS at six months were virtually the same in both groups: 3 (1, 4) for oxygen and 3 (1, 5) for control. From the ordinal regression analysis, the covariate-adjusted odds ratio was 1.04 (95% CI 0.67, 1.60); this favoured the oxygen group, indicating that the odds for a better (i.e. lower) score on the mRS were 4% higher in the oxygen group than in controls, but the difference was non-significant (*p* = 0.86). The corresponding odds ratio without adjustment was 0.95 (95% CI 0.62, 1.46; *p* = 0.82). The distribution of scores on the mRS is shown in Figure 2. While there was no difference between the groups for the milder mRS Scores (0–2), there appeared to be a shift at the more severe end, with fewer patients in the oxygen group being totally dependent (mRS of 5).

### The Barthel Index

This assessment was completed for 114 (77%) and 113 (80%) participants in the oxygen and control groups respectively. The median (IQR) score on the 3-point Barthel was 6 (5, 8) for the oxygen and 7 (6, 8) for the control group. Using the conversion formula (see Methods), the median scores on the 20-point scale were 14 and 17, respectively. The majority of respondents were fully continent (60 [53%] vs. 72 [64%]), independent in bed to chair transfers (71 [62%] vs. 81 [72%]) and able to able to mobilize independently (58 [51%] vs. 70 [62%]), for oxygen and control respectively (see Table 3). The covariate-adjusted odds ratio from the ordinal regression analysis was 1.50 (95% CI 0.94, 2.37); this favours the control group, indicating that the odds for a worse (i.e. lower) score on the Barthel Index were 50% higher in the oxygen group than in controls, but not significantly (*p* = 0.09). The corresponding unadjusted odds ratio was just significant: 1.54 (95% CI 1.00, 2.38; *p* = 0.05).

**Table 3:** The abbreviated Barthel Index.

		Oxygen	Control
		*n* = 113	*n* = 114
Bladder	0 Catheterized^a^	6 (5)	8 (7)
	0 Incontinent^a^	17 (15)	11(10)
	1 Occasional accident	31 (27)	22 (20)
	2 Continent	60 (53)	72 (64)
Transfers	0 Unable – no sitting balance	8 (7)	4 (3)
(bed/chair)	1 Major help needed	14 (12)	13 (12)
	2 Minor help needed	21 (18)	15 (13)
	3 Independent	71 (62)	81 (72)
Mobility	0 Immobile	12 (11)	5 (4)
	1 Wheelchair independent	12 (11)	7 (6)
	2 Walks with help of one person	32 (28)	31 (27)
	3 Independent	58 (51)	70 (62)

Data are number (%). Only participants providing data on all three points are included.

a Catheterized and incontinent are indicated separately, but each was scored as 0.

### The Nottingham Extended Activities of Daily Living Scale

This assessment was completed for 93 (63%) and 100 (71%) participants in the oxygen and control groups respectively. The covariate-adjusted means in the oxygen and control groups were 47.62 and 49.23 respectively, giving an adjusted mean difference (control minus oxygen) in ability scores of 1.59 (95% CI −3.26, 6.45), in favour of the oxygen group. This difference was not significant (*p* = 0.52). The mean unadjusted scores for the oxygen and control groups were 48.39 and 48.47 respectively, giving a mean difference of −0.08 (95% CI −5.34, 5.51; *p* = 0.98). Combining scores for all 20 tasks, the mean percentage of subjects able to perform the activities alone was 55% in the oxygen group and 58% in the control group (Table 4).

**Table 4:** Nottingham Extended Activities of Daily Living.

	Oxygen (*n* = 93)	Control (*n* = 100)
**Mobility**		
Walk around inside	65 (70)	73 (73)
Climb stairs	50 (54)	59 (59)
Get in and out of car	43 (46)	63 (63)
Walk on uneven ground	43 (46)	56 (56)
Travel on public transport	30 (32)	32 (32)
Drive a car	32 (36)	39 (39)
**Eating and Drinking**		
Manage to feed yourself	78 (84)	88 (88)
Manage to make a hot drink	64 (69)	79 (79)
Take hot drinks from 1 room to another	58 (62)	68 (68)
Do the washing up	57 (61)	70 (70)
Make yourself a hot snack	59 (64)	68 (68)
**Money and Shopping**		
Manage own money	57 (61)	61 (61)
Do own shopping	31 (33)	37 (37)
**Housework**		
Wash small items of clothing	51 (55)	54 (54)
Do a full clothes wash	45 (48)	48 (48)
**Leisure & Communications**		
Read newspapers & books	74 (80)	80 (80)
Use the telephone	73 (79)	81 (81)
Write letters	47 (51)	48 (48)
Go out socially	39 (42)	32 (32)
Manage own garden	24 (26)	24 (24)

Data are number (%) of participants able to perform the activity alone (easily or with difficulty). Only participants providing data on all 20 items are included.

### Quality of life (EuroQol)

EQ-5D scores were obtained for 110 (74%) and 112 (79%) participants and EQ-VAS scores for in 81 (55%) and 96 (68%) participants, in the oxygen and control groups respectively. Scores on the individual dimensions are summarized in Table 5. The covariate-adjusted mean EQ-5D utility scores in the oxygen and control groups were 0.50 and 0.49 respectively, giving an adjusted mean difference (control minus oxygen) in utility scores of −0.01 (95% CI −0.10, 0.07), in favour of the oxygen group (*p* = 0.79). The unadjusted mean scores for the oxygen and control groups were 0.50 and 0.50 respectively, giving a mean difference of 0.00 (95% CI −0.09, 0.09; *p* = 0.99).

**Table 5:** Quality of life.

		Oxygen	Control
	Responses	*n* = 110	*n* = 112
Mobility	No problem	34 (31)	30 (27)
	Some problems	69 (63)	74 (66)
	Not able	7 (6)	8 (7)
Self-care	No problem	59 (54)	60 (54)
	Some problems	41 (37)	35 (31)
	Not able	10 (9)	17 (15)
Usual activities	No problem	40 (36)	44 (39)
	Some problems	36 (33)	42 (38)
	Not able	34 (31)	26 (23)
Pain	No pain	43 (39)	40 (36)
	Some pain	59 (54)	67 (60)
	Extreme pain	8 (7)	5 (4)
Anxiety	No anxiety	46 (42)	43 (38)
	Some anxiety	57 (52)	62 (56)
	Extreme anxiety	7 (6)	7 (6)

Scores for the five dimensions of the EuroQol. Data are numbers (%). Only participants providing data on all five dimensions are included.

The covariate-adjusted mean scores for the EQ-VAS were 59.24 and 54.84 for the oxygen and control groups respectively, giving an adjusted mean difference (control minus oxygen) of −4.40 (95% CI −11.43, 2.63), in favour of the oxygen group (*p* = 0.22). The corresponding unadjusted means scores were 58.85 and 55.33 giving a mean difference of −3.52 (95% CI −10.88, 3.85; *p* = 0.35); owing to the high rate of missing data (39%) for the EQ-VAS, these estimates should be interpreted with caution.

### Six-month survival

**Figure 3:**
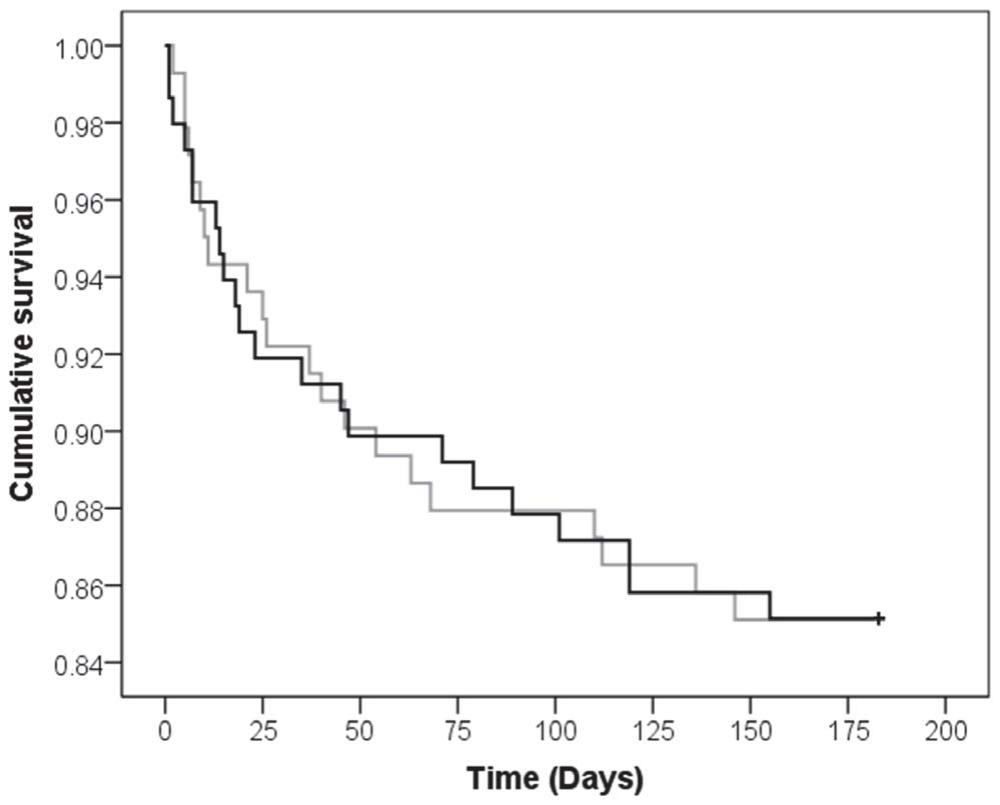
Cumulative survival in the oxygen (black line) and control (grey line) groups at six months.

At six months, 22 (15%) patients had died in the oxygen group and 21 (15%) had died in the oxygen group. Mean survival time at six months was 162 days for both groups (log-rank test, *p* = 0.99); see Figure 3. In the adjusted analysis using Cox regression, the hazard function was 1.10 (95% CI 0.59, 2.07), suggesting that those in the control group had a slightly higher probability of dying at a given time; however, this hazard ratio was non-significant (*p* = 0.76).

### Memory

Memory was reported as “as good as before the stroke” in 51 (53%) and 46 (45%) and as “worse than before the stroke” in 45 (47%) and 56 (55%) of the 96 and 102 respondents to this question respectively in the oxygen and control groups (exact *χ* ^2^ = 1.28, *p* = 0.32).

### Place of residence

At six months 98 (83%) and 104 (92%) of the 113 and 118 respondents in the oxygen and control groups respectively resided in a private house (alone or with relatives), 19 (16%) and 8 (7%) were in an institution (nursing home or residential home), and 1 (1%) and 1 (1%) were still in hospital (exact *χ* ^2^ = 4.55, *p* = 0.08).

### Sensitivity analyses

A sensitivity analysis using only participants with complete follow-up data was performed for the mRS, the Barthel Index, the NEADL, and the EuroQol. These analyses produced very similar estimates to the intention-to-treat analyses, and the statistical conclusions were unchanged (see Table 6).

**Table 6:** The sensitivity analysis.

	Intention-to-treat analysis			Complete case analysis		
	Estimate (95% CI)	*p* value	*n*_1_, *n*_2_ a	Estimate (95% CI)	*p* value	*n*_1_, *n*_2_ a
Modified Rankin Scale^b^	1.04 (0.67, 1.60)	0.86	148, 141	1.07 (0.68, 1.67)	0.78	126, 130
Barthel Index^b^	1.50 (0.94, 2.37)	0.09	148, 141	1.50 (0.92, 2.45)	0.11	114, 113
NEADL Scale^c^	1.59 (−3.26, 6.45)	0.52	148, 141	0.64 (−3.71, 4.99)	0.77	93, 100
EuroQol EQ-5D^c^	−0.01 (−0.10, 0.07)	0.79	148, 141	−.004 (−0.09, 0.10)	0.94	110, 112
EuroQol EQ-VAS^c^	−4.40 (−11.43, 2.63)	0.22	148, 141	−2.35 (−9.54, 4.84)	0.52	81, 96

a Numbers for oxygen and control groups, respectively;

b odds ratio (reference category is oxygen);

c mean difference (control–oxygen); CI = confidence interval.

Higher scores on the modified Rankin scale and on the Nottingham Extended Activities of Daily Living (NEADL) Scale are worse; higher scores on the Barthel Index and EuroQol are better.

## Discussion

The main finding of this pilot study is that supplemental oxygen given for 72 h after stroke is safe, but has no large effect on measures of the level of disability, the ability to perform basic and extended activities of daily living, quality of life, and place of residence at six months.

Rønning et al have previously shown a similar lack of benefit in terms of survival and the level of independence in a quasi-randomized study of low-flow oxygen supplementation at a rate of 3 L/min for 24 h in a sample of 550 stroke patients recruited within 24 h of symptom onset [22]. Subgroup analysis in this study suggested possible harm in patients with mild stroke, and potential benefit in severe stroke. Our pilot study is not powered to exclude or confirm a differential effect dependent on stroke severity. A very small pilot study by Singhal et al of high-flow oxygen treatment at a rate of 45 L/min for 8 h via face mask in 16 patients within 12 h after acute stroke showed transient improvements in cerebral blood volume and blood flow within the ischaemic regions with hyperoxia (higher than normal blood oxygen concentrations), but no long-term clinical benefit at three months [23]. This study was followed by a larger trial of the same intervention, which was stopped after 86 patients were enrolled because of an imbalance of deaths in favour of the control group (20% on oxygen vs. 8% on room air). The results are available on the clinicaltrials.gov website [24], but not published in a journal. This study raises the concern that not only too low, but also too high oxygen concentrations could be detrimental. A similar adverse effect of hyperoxia on mortality has been suggested in a recent meta-analysis (*n* = 387) of oxygen treatment (4–6 L/min) in myocardial infarction [25] and in a large (*n* = 6326) observational study of cardiac arrest survivors in the intensive care unit, where hyperoxia (PaO_2_ of 300 mmHg [40 kPa] or greater) increased the odds of in-hospital death by 1.8, with higher mortality in hyperoxic than in hypoxic (PaO_2_ of less than 60 mmHg [8 kPa]) patients [26]. In contrast to these studies, the dose of oxygen given in the Stroke Oxygen Pilot Study aims to maintain normoxia, rather than achieve hyperoxia. In the subsequent main study (the Stroke Oxygen Supplementation Study: SO_2_S) [27], we included a third arm, where oxygen is only given at night, at a time when the risk of hypoxia is highest [28], thus reducing the chance of hyperoxia in the day, when oxygen concentrations are more likely to be normal.

We previously reported that routine oxygen supplementation given for 72 h at a rate of 2 or 3 L/min, dependent on baseline oxygen saturation, lead to a small, but statistically significant improvement in neurological recovery at one week [7]. While neurological recovery (i.e. the difference between one-week and baseline NIHSS scores) was better, NIHSS scores at one week were the same in both groups. As NIHSS at one week is a strong predictor of long-term functional outcome [29]–[31], it is not surprising that we found no difference in the mRS at six months. However, baseline NIHSS was imbalanced in this small pilot study with a higher (worse) NIHSS score in the oxygen group. Patients in the oxygen group were older, and there were more females, and this is reflected in lower probability of being free from dependency at six months on the SSV predictive model. When the six-month mRS is adjusted for covariate imbalance, oxygen treatment confers a small (4%), statistically non-significant increase in the odds of achieving a better (lower) mRS score. As oxygen is a safe and cheap treatment option, which is available even in very small hospitals and pre-hospital health settings throughout the world, even very small differences in outcome could have a significant impact on stroke outcome worldwide. A very large study is required to test a small, but worthwhile effect. This is now on-going (SO_2_S) [27].

Oxygen was only given for 72 h, and while this is longer than in the two earlier studies, it could be difficult to discern an effect at six months, long after the end of the intervention. SO_2_S therefore has the main outcome assessment at 90 days, an assessment time point now suggested as appropriate for acute interventions, but will also follow patients at 6 and 12 months to see if any effect persists long-term. The mRS at three months or later has recently been suggested as the preferred outcome measure by the European Stroke Organization Outcomes Working group, with other outcome scales used as supporting scales to corroborate the results [32]. Findings of the mRS and the other outcome scales in this study were consistent, supporting validity of the findings. From a statistical point of view, the additional scales could be considered redundant, as they do not contribute to the assessment as to whether the treatment is effective or not. They do, however, put the results into a ‘real’ life context, and contain information that stroke survivors consider important [33].

In this trial we assessed six-month outcome by questionnaire. There were differing rates of missing data on the outcome measures. The rate tended to be higher for measures that required a direct response from the participant (e.g. the EuroQol; 23% missing for the EQ-5D, 39% for the EQ-VAS) and/or were lengthy or complex to complete (e.g. the NEADL; 33% missing). The primary outcome measure, the mRS, is a simple scale and can if necessary be completed by a third party or from information in the medical notes; accordingly, the rate of missing values on this measure was relatively low (11%). Those with responses on the mRS did not differ significantly from those with missing values in terms of sex (*p* = 0.90), age (*p* = 0.19), or probability of being from from dependency at 6 months (*p* = 0.10), and although the rate of missing values was higher in the oxygen group (15%) than in the control group (8%), this difference was non-significant (*p* = 0.06). This provides some reassurance that the pattern of missing data on the primary outcome was not systematic. A number of steps were taken to establish the primary outcome (mRS) where it was initially unavailable – by resending questionnaires that had not been completed, by consulting follow-up records within the hospital information system and patient notes, and by enquiries to the patient's general practitioner, wherever possible. These strategies have been incorporated and strengthened in the protocol for the main study [27]. Patients who changed their address and general practitioner, and who were not readmitted to the hospital, could not be followed, as we had no access to the follow up address. Missing values were higher (from 21% to 39%) on other outcomes, suggesting that these should be interpreted with caution. Follow-up rates are lower than in recently published large multicentre trials such as the CLOTS trial [34], the COSSACS study [35], and IST-3 [36]. The SOS study was an unfunded pilot, which had insufficient staff to systematically track non-responders. A recent study has shown that response rates can be greatly improved with good levels of intermodality agreement by telephone follow-up of non-responders [37]; this strategy has been included in the protocol for the main study. Only just over a third of patients completed the questionnaires unaided, in a further third it was done by, or with the help of, a friend or relative. The SOS pilot study has therefore helped us to refine follow up procedures for SO_2_S, with computer generated prompts for follow up, clear guidance on the questionnaire that help of a relative or friend with completion is acceptable, regular review of response rates, and back-up telephone calls for repeated non-responders.

The sample size of this pilot study was the maximum attainable in relation to available resources, and was not the result of a sample size calculation. Accordingly, the analyses were not formally powered, and it should in particular be noted that the ratio of cases to events in the Cox regression analysis may have resulted in some overfitting. The sample size for the main study [7] has been determined through a formal power calculation.

In conclusion, the results of this pilot study do not show a benefit on long-term outcome, but suggest the possibility a small improvement in functional outcome when corrected for imbalance in baseline prognostic factors. It confirms that the duration and dosing schedule of oxygen in this study are safe. The results have helped to shape a larger, fully powered study of oxygen supplementation after acute stroke, which is now in progress.

## Supporting Information

Checklist S1 CONSORT checklist.
(DOC)

Protocol S1 Trial protocol.
(DOC)
